# Tackling malaria transmission at a single cell level in an endemic setting in sub-Saharan Africa

**DOI:** 10.1038/s41467-022-30268-w

**Published:** 2022-05-13

**Authors:** Antoine Dara, Sunil Kumar Dogga, Jesse Rop, Dinkorma Ouologuem, Fatalmoudou Tandina, Arthur M. Talman, Abdoulaye Djimdé, Mara K. N. Lawniczak

**Affiliations:** 1 Malaria Research and Training Centre (MRTC), Faculty of Pharmacy, Université des Sciences, des Techniques et des Technologies de Bamako (USTTB), Point G, P.O. Box, 1805 Bamako, Mali; 2 Wellcome Sanger Institute, Hinxton, CB10 1SA UK; 3 MIVEGEC, IRD, CNRS, University of Montpellier, Montpellier, France

**Keywords:** Parasite genomics, Antimicrobial resistance

## Abstract

Studying malaria transmission biology using scRNA-sequencing provides information on within-host strain diversity and transcriptional states. Here, we comment on our collaborative efforts at establishing single-cell capacities in sub-Saharan Africa and the challenges encountered in Mali’s endemic setting.

## The burden of malaria

Although malaria is a treatable and preventable disease, an estimated 241 million cases and 627,000 deaths were recorded in 2020, 14 million more cases and 69,000 more deaths compared to 2019^1^. This increase reflects another impact of the COVID-19 pandemic, which curtailed effective delivery of malaria services and caused huge disruptions in control and elimination programs worldwide. Malaria control efforts remain critical, having averted an estimated 1.7 billion cases and 10.6 million deaths in the period 2000–2020^1^. Using novel approaches to better understand parasite transmission can only improve our ability to control malaria, and thus we have set out to study transmission dynamics in Mali, where malaria remains a serious burden.

## Understanding *P. falciparum* transmission

Of the five human malaria-causing species, *Plasmodium falciparum* is currently responsible for over 99% of cases in the WHO African region^1^. Genetic variation within *P. falciparum* is high and the parasite population existing in one infected person can contain many diverse strains, with potentially varying virulence and pathogenicity^2–5^. This complexity can be generated both by co-transmission of (often related) parasites in a single infectious bite and by superinfection caused by bites from multiple independent mosquitoes. Complex infections with more than a single parasite strain occur in more than 70% of infections in endemic populations^6^. Additionally, the genetic diversity of parasite populations varies across seasons and regions, challenging effective disease management and control. In light of this, understanding and monitoring parasite diversity and transmission in response to environmental and interventional pressures are necessary to design control strategies, especially in the face of increasing resistance to antimalarial drugs. The importance of such surveillance has been starkly demonstrated in detecting variants of SARS-CoV-2 around the world, which has in turn informed public health policy.

## Tackling imminent questions with single-cell technologies

Strategies for genotyping parasite diversity within malaria-infected people are typically based on gene-specific PCR-based typing, microsatellite analysis, SNP-barcoding, targeted and genome-wide sequencing^7^. These methods assess complexity of infection by evaluating variation at select highly variable loci. However, they are limited in their ability to understand what strains—i.e. distinct haplotypes across many loci—are present and whether each strain is represented by both asexual stages (actively replicating forms responsible for malaria disease symptoms) and/or sexual stages (non-replicating female and male gametocytes that are responsible for malaria transmission via mosquitoes). This information is essential when assessing mixed infections in the context of virulence, transmission, and the spread of drug resistance. For example, if drug-resistant parasites are detected in an assay, but not producing sexual transmissible forms, this is highly relevant information.

Our study to better understand transmission dynamics is a close collaboration of Dr. Mara Lawniczak’s group at the Wellcome Sanger Institute and Professor Abdoulaye Djimdé’s group at the Malaria Research and Training Center (MRTC) in Mali. We are harnessing single-cell transcriptomics to understand fundamental aspects of parasite transmission biology by analyzing the populations of malaria parasites within individual people from an endemic area. Our field site is based in Faladie, a small village 77 km northwest of the Malian capital, Bamako. Faladie is characterised by hyper-endemic seasonal malaria with three human *Plasmodium* species—*P. falciparum*, *P. ovale* and *P. malariae*. Our project is funded by the Medical Research Council in the United Kingdom, which funds our research collaboration and contributed to improved research infrastructure through the building of a permanent insectary containing clean laboratory space and reliable power supply next to the clinic in Faladie. Furthermore, we also received generous support from 10X Genomics towards the installation of a Chromium 10X controller in Bamako—the first in West Africa—enabling in-country single cell studies. These long term investments will facilitate high quality research that we and other scientists, both local and foreign, undertake in Mali.

Our study is generating single-cell transcriptional atlases of the three main malaria species from infected individuals. Outputs from our endeavour will be rapidly added to the Malaria Cell Atlas website, which already serves as a valuable data resource for the malaria research community but currently focuses on cultured lab strains. We will explore within and between-host diversity of sexual and asexual populations of *P. falciparum* from 50 symptomatic and 50 asymptomatic carriers. Single-cell RNAseq allows us to evaluate transcriptional patterns of parasites in different contexts—we can use the data to simultaneously assign the specific life cycle stage and sex (where relevant) as well as the strain genotype to every single parasite we investigate through the use of allelic information contained within each cell’s transcripts^8^. This provides information on the genetic diversity of circulating parasites, enabling us to evaluate the relatedness of asexual parasites and female and male gametocytes within an infection, and how this compares within and between hosts experiencing different clinical outcomes. The transmission of sexual stages to mosquito vectors will also be explored to understand the dynamics of reproduction and recombination within this infectious reservoir. The global output of this work should greatly advance our understanding of *Plasmodium* biology.

## Many challenges to overcome

Undertaking such a study in a field setting like Faladie, where there is no stable electricity and running water, comes with its share of challenges. We briefly discuss the major hurdles that researchers in sub-Saharan Africa (sSA) encounter, including challenging infrastructure, unreliable supply chain for reagents and consumables, and poor access to maintenance services for research equipment required to increase sequencing capacity in Mali.

### Infrastructure lacking at a larger scale

Given the remote location of field sites with high malaria prevalence, transportation for long distances on poor road conditions is necessary. Also, depending on the study, reliable temperature-controlled sample storage facilities are vital. Due to erratic electricity supply, our clinic site currently relies on solar power, which can be unsustainable, especially during the rainy season. Though backup generators provide a fix, they can be unreliable and incur massive costs. Transportation of biological samples is similarly complicated by long distances and extremes of the rainy season’s road conditions, temperature and humidity.

### Unreliable supply and maintenance of research resources

Another burning issue that researchers in Mali and sSA constantly face is timely and reliable access to consumables and reagents. Obtaining reagents can take at least 3–4 months, an optimistic estimate, from the day of purchase, which often occurs through local distributors charging much more than the manufacturer’s price. This situation has been exacerbated during the COVID-19 pandemic. Therefore, researchers often rely on international collaborators to receive consumables in time^9^.

### Underdeveloped local NGS capacity

Our current study involves shipping processed cDNA samples to the Sanger Institute for sequencing, adding costs associated with shipment as well as sample transportation risk. We envision a prospect where local sequencing capacity will be bolstered to support the processing of samples from blood draw to library preparation to sequencing to quality control and data analysis. There is a sequencing facility at MRTC in Bamako, with access to an Illumina MiSeq instrument, a SeqStudio instrument, and the 10X Chromium controller. A server with 140 terabyte storage capacity is available, dedicated to bioinformatics analyses. Local sequencing capacity is still in its infancy in Mali, and massive investments are needed to expand these capabilities. This is challenged by the requirement of expertise in using these sophisticated instruments, which is compounded by the high cost of their maintenance and consumable supply chain issues.

## Addressing the challenges

Advanced technology such as single-cell sequencing involves high equipment and maintenance costs, and hence is out of reach for most laboratories in sSA. MRTC is to our knowledge the only research centre that has access to 10X single-cell technology in West Africa. Thus, it can serve as a hub for West African researchers to perform single-cell sequencing experiments. More regional research centres need to be established, which can be equipped with such infrastructure acting as focal points for smaller laboratories in surrounding regions. These research centres can collaborate and negotiate contracts with manufacturers to address local needs and technical services. Furthermore, great initiative is needed from technology-providing companies to increase their presence in sSA, considering research impact on public health as well as commercial incentives expected from burgeoning Africa-led research—recent increase in Africa-led projects like the ambitious three million African genomes project being an example^10–12^. Roche and 54Gene are increasing their investments to accelerate genetics research and diagnostics development in the continent. With malaria mRNA vaccines being a possibility in the near future, Pfizer BioNTech and Moderna are advancing plans to set up manufacturing plants in Rwanda, Senegal and Kenya. Such regional manufacturing capacity and distribution hubs together with technical support services are in the right direction towards facilitating local research^13,14^.

Despite the above-mentioned challenges, the first SARS-CoV2 genome sequenced in Mali was generated locally with great difficulty and ingenuity^15^. Such local research output is instrumental to inspire more researchers and boost public trust. The goal should be to provide an environment where Africa-led research takes an active lead in the fight against malaria, with independence and collaboration. Our collaborative study is a small step towards realising such a future where Africa sets its own research agenda and addresses health challenges on its own capacity.
